# Neural patterns of threat response in adolescents predict vulnerability for and resilience against internalizing symptoms during COVID-19 waves

**DOI:** 10.1016/j.ynirp.2023.100177

**Published:** 2023-05-29

**Authors:** Anna Tyborowska, Yvonne van den Berg, Mahur M. Hashemi, Hannah C.M. Niermann, Antonius H.N. Cillessen, Ivan Toni, Karin Roelofs

**Affiliations:** a Behavioural Science Institute, Radboud University, Thomas van Aquinostraat 4, 6525 GD, Nijmegen, the Netherlands; b Donders Institute for Brain, Cognition and Behaviour, Radboud University, Kapittelweg 29, 6525 EN, Nijmegen, the Netherlands

**Keywords:** Threat reactivity, Fight-flight response, Amygdala, Stress-related symptoms, COVID-19, Vulnerability, Resilience

## Abstract

Defensive stress reactions, such as freezing and active fight-or-flight, are relevant for coping with threat. Action-preparatory activity supporting these reactions, including the amygdala, has been posited as a potential marker for stress-resilience. We considered the successive COVID-19 lockdowns as two pervasive stressors, to prospectively investigate the predictive value of neural threat-responses towards symptom development. Five years prior to the COVID-19 pandemic, 17-year-old adolescents (n = 64, Baseline-17) performed the fMRI-adapted Go/Nogo Under Threat (GUNT) task, where threat-anticipatory freezing reactions and transition to action are evoked to avoid a shock. A majority (n = 44) made themselves available for follow-up assessments before COVID (Baseline-20, age 20), during the first COVID-19 lockdown in the Netherlands (LD1, age 22.5), and during a second lockdown (LD2, age 23). The GUNT task quantified neural (thalamic, subcortical, amygdala) and physiological (bradycardia) markers of threat-anticipatory freezing and transition to action (mediated by anterior cingulate cortex). Threat-anticipatory amygdala responses (Baseline-17) were linked to stressor resilience, as quantified by self-reported anxiety symptoms between LD1 and LD2. However, stronger amygdala responses to low threat cues (Baseline-17) were associated with stronger anxiety symptoms. These effects occurred over and above early-life stress, COVID-19 stress burden, and overall symptom changes between age 17 and 20. These findings suggest that amygdala responses to acute threat provide a marker for resilience against real-life stressors, with adequate threat discrimination signaling resilience and stronger amygdala responses to low threat predicting vulnerability. The findings support the notion that neural responses to threat are instrumental for adaptive coping with pervasive stress.

## Highlights

• Adolescent neural threat-response is related to anxiety symptoms during adulthood.
• Threat-induced amygdala reactivity predicts resilience to anxiety during COVID-19.
• Higher amygdala responses to low threat predict anxiety symptoms during COVID-19.
• Differentiation of threat and safety cues may signal resilience to later stress.

## Graphical abstract

**Figure:**
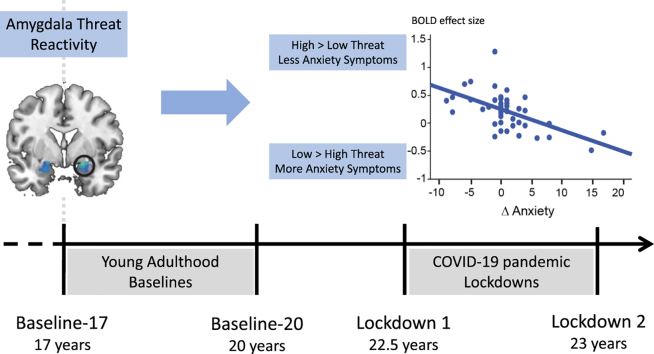

## 1 Introduction

Stress-related disorders, such as anxiety, depression, addiction or post-traumatic stress disorder, are thought to be triggered or exacerbated by stressors such as traumatic events, life transitions, or emotionally challenging situations (Chaby et al., 2017; Kalisch et al., 2017). Individual differences in acute defensive threat-reactivity have been implicated in internalizing psychopathologies (Everaerd et al., 2015; Geng et al., 2018; Hashemi et al., 2021; Hulsman et al., 2021; Niermann et al., 2018; Shackman et al., 2017). Isolating adolescent-specific neural profiles of threat reactivity is of particular importance for understanding vulnerability and resilience factors – especially given the onset of affective disorders, such as anxiety, peaking in puberty (Kessler et al., 2007). We tested neural, physiological, and behavioral correlates of threat-induced anticipatory responses in adolescence to identify potential resilience markers against the development of stress-related symptomology.

A fundamental, cross-species response to threat is an automatic defensive cascade of freezing and fight/flight reactions (Blanchard, 2017; Fanselow and Lester, 1988; Gabrielsen et al., 1985; Kempster et al., 2013). Threat-induced anticipatory freezing is supported by the parasympathetic branch of the autonomic nervous system, providing a temporary break on the motor system and a net heart rate deceleration, or threat bradycardia (Fanselow, 1994; Schenberg et al., 1993). Indeed, threat-induced bodily immobility is accompanied by bradycardia in both animals and humans (Roelofs and Dayan, 2022). Bradycardia and bodily freezing are important for action preparation and both coincide with a startle response to threat (Szeska et al., 2021; van Ast et al., 2022; Wendt et al., 2017). When assessing humans in the MRI scanner, bradycardia is taken as a proxy of freezing (Hashemi et al., 2019; Hermans et al., 2013; Schipper et al., 2019).

In both animals and humans, freezing, is organized through neural circuits supporting defensive behavior, namely the amygdala-periaqueductal grey (PAG) and medial prefrontal cortex network (Blanchard et al., 2003; Fanselow, 1994; Hashemi et al., 2019; Korte et al., 2005; Roelofs, 2017; Tovote et al., 2016). The amygdala is involved in the switch between defense modes from freeze to active fight/flight responses, via its connections with the PAG, medial prefrontal cortex (mPFC) and anterior cingulate cortex (ACC; Gozzi et al., 2010; Hashemi et al., 2019; Schipper et al., 2019; Tovote et al., 2015). Amygdala projections to the dorsolateral (dl)PAG activate flight/flight responses. On the other hand, during freezing, projections from the central nucleus of the amygdala to GABAergic ventrolateral (vl)PAG interneurons disinhibit projections to medulla and spinal cord motor neurons, thus resulting in immobility (Keay and Bandler, 2001; Tovote et al., 2016; Walker and Carrive, 2003). During threat anticipatory freezing, concurrent bradycardia is due to the vlPAG also activating the vagal pathway (via the nucleus ambiguous) to generate parasympathetically-driven heart rate deceleration (Farkas et al., 1997; Walker and Carrive, 2003). Immobility and bradycardia during freezing, associated with sympathetically driven sensory processing and action preparation, lead to an alerted state poised for a response (Roelofs and Dayan, 2022; Skora et al., 2022).

Neural, behavioral, and physiological responses to acute threat are relevant for dealing with an immediate challenge. Freezing supports decision-making by facilitating action preparation (Gladwin et al., 2016; Hashemi et al., 2019; Klaassen et al., 2021), perception (de Voogd et al., 2022; Lojowska et al., 2015, 2019), and integration of outcome values serving subsequent approach-avoidance decisions (Klaassen et al., 2021; Livermore et al., 2021). Indeed, threat anticipatory freezing has been shown to be relevant for active coping (Hashemi et al., 2019, 2021). However, altered threat-related responses may also reflect intrinsic vulnerabilities related to stress coping. At the neural level, heightened threat-induced amygdala reactivity has been proposed as a phenotypic risk factor for the development of internalizing psychopathology (Everaerd et al., 2015; McLaughlin et al., 2014; Shackman et al., 2017; Swartz et al., 2015). Therefore, deviations in both extremes (i.e., hyper- and hypo-responding to threat) may pose a risk for psychopathology and be specific for a particular developmental or lifespan stage. Behaviorally, both excessive and blunted freezing to acute threat have been linked to increased vulnerability to internalizing symptoms in early and late adolescence, respectively (Niermann et al., 2018), a finding further replicated for blunted freezing (Held et al., 2022). These findings highlight the adaptive role of both freezing and threat anticipatory responses for active coping, with a link to vulnerability when those responses deviate (see also Roelofs and Dayan, 2022).

A long-lasting and unprecedented threat that has resulted in dramatic changes in everyday life has been the COVID-19 pandemic. Emerging evidence suggests that the pandemic, similar to other widescale and global societal-level disruptions (i.e., natural disasters or war), has also contributed to the development of stress-related psychopathology (McLaughlin et al., 2022). Numerous studies have reported an increase in mental health problems, such as anxiety and depression during the initial COVID-19 lockdowns and throughout the course of the pandemic, in adolescents (Gotlib et al., 2022; Hussong et al., 2021; Liu et al., 2022; Magson et al., 2021) as well as in emerging adults (Afifi et al., 2022; Graupensperger et al., 2022; Hawes et al., 2022; Kornilaki, 2022; Kujawa et al., 2020; Liu et al., 2020; Shanahan et al., 2022; Skinner et al., 2022; Sun et al., 2021; van den Berg et al., 2021). Only a handful of neuroimaging studies have examined whether neural activity assessed before the Covid-19 lockdowns predicted mental health outcomes during the pandemic (adolescence overall – Miller et al., 2021; Perica et al., 2021; early/middle adolescence - Weissman et al., 2021; late adolescence/emerging adulthood – Gotlib et al., 2022; Khorrami et al., 2022; Perica et al., 2021). However, most studies did not assess amygdala responses to threatening versus safe cues, except Weissman et al., 2021. The latter study in early and middle adolescents, demonstrated that decreased amygdala responses to negative stimuli was linked to stronger stress-related internalizing symptoms at follow-up during the COVID-19 pandemic (Weissman et al., 2021). This suggests that better differentiated threat responses during adolescence may be a protective factor, particularly in instances of pervasive stress and uncertainty.

Here, we evaluated this hypothesis further by assessing the role of adaptive stress-responses in late adolescence. Late adolescence is a critical developmental window when neural circuits involved in control of automatic defensive tendencies continue to mature (Herting et al., 2018; Mills et al., 2016; Tamnes et al., 2017; Zimmermann et al., 2019). Especially relevant for threat-related neural responses, late adolescence marks a discrete window of reorganization, when prefrontal inputs to the amygdala undergo pruning (Cressman et al., 2010; Maithe Arruda-Carvalho et al., 2017). In this study, we considered reactivity to high threat and low threat situations in the context of active coping. These conditions were operationalized in the fMRI-adapted Go/Nogo Under Threat (GUNT) task, in which threat-anticipatory freezing reactions and transition to action are evoked to avoid a shock (Hashemi et al., 2019).

This study had two goals. First, we aimed to replicate previously identified neural and physiological responses related to freezing and the subsequent switch to action in an adolescent sample. Second and foremost, we aimed to evaluate whether these neural defensive responses provide markers for chronic stress-related symptoms. Specifically, we tested whether action-preparatory threat responses in core regions of the neural defense system (amygdala and PAG) were linked to resilience or vulnerability to negative effects of a long-lasting and pervasive stressor, namely the COVID-19 crisis. We differentiated chronic stress effects related to *changes* in general psychological distress, anxiety, and depression occurring between the first and second COVID-19 lockdowns in the Netherlands, from *acute* responses to each lockdown. We tested two alternative hypotheses for action-preparatory responses in late adolescence. In line with the phenotypic risk premise (e.g., Shackman et al., 2017), heightened threat-induced anticipatory amygdala and PAG activity would be related to more internalizing symptoms during the COVID-19 pandemic. Alternatively, in line with the adaptive value of preparatory responses, threat-related amygdala and PAG activity could be a resilience factor against the development of later internalizing symptoms. Additional exploratory analyses examined associations with symptoms across the whole brain, as well as in circuits facilitating the switching to action. Following recommendations to isolate vulnerability and resilience factors to challenging and stressful events (Kalisch et al., 2017), we controlled for developmental changes in symptom levels pre-pandemic, across late adolescence and in young adulthood as well as early-life events (until age 5) – important transition periods for mental health outcomes (Tyborowska et al., 2018; Zahniser and Conley, 2018).

## 2 Materials and methods

### 2.1 Participants and recruitment

All actively participating 17-year-old adolescents from the Nijmegen Longitudinal Study on Child and Infant Development (NLS; *n* = 116) were approached to take part in the imaging assessment at Baseline-17 (NLS Wave 10). The NLS participants were recruited from a range of social economic backgrounds, which is representative of the Dutch population of families with children in the same age range. For more information on the NLS sample and social economic status see (Smeekens et al., 2007; Tyborowska et al., 2018; Van Bakel and Riksen-Walraven, 2002; van den Berg et al., 2021). Functional magnetic imaging was obtained from 68 participants who met standard MRI inclusion criteria. Participants who did not follow task instructions (*n* = 3) or moved excessively during scanning (*n* = 1) were excluded (Supplementary Material S2), resulting in 64 participants (36 boys; sex assigned at birth). All participants had normal or corrected-to-normal vision, no history of psychiatric disorders or neurological illness (as indicated by parent/guardian report).

**Fig. 1:**
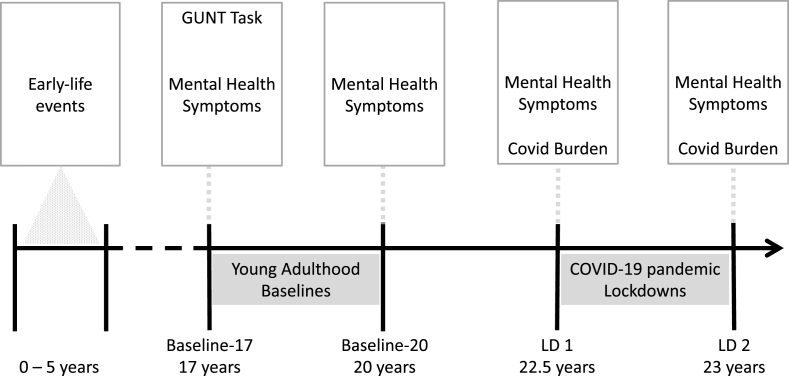
**Study timeline and measurements.** The Go/Nogo Under Threat Task (GUNT) was assessed in late adolescence at Baseline-17. Mental health symptoms refer to anxiety, depression, and psychological distress. The time-period between Baseline-17 and Baseline-20 provides a baseline for developmentally occurring symptom changes during young adulthood. Increases in symptoms between Lockdown 1 (LD1) and Lockdown 2 (LD2) capture effects of chronic stress related to the COVID-19 pandemic, while measurements at LD1 and LD2 capture acute stress responses. Negative events occurring in the first 5 years of life were prospectively assessed and used as a measure of early-life stress.

Three online follow-up assessments (see section 2.4 Mental Health) were carried out at age 20 (Baseline-20, n = 98), during the first COVID-19 lockdown in the Netherlands (LD1, age 22.5, n = 86), and second lockdown (LD2, 6-months later, n = 91)(Fig. 1). As in previous measurement rounds of the NLS, data from Baseline-17 and Baseline-20 was collected around the time of the participants’ birthdays. At LD1 and LD2, all participants were assessed in the same weeks, during which COVID-19 restrictions were initiated by the Dutch government due to the rapid rise in infections (social distancing of 1.5 m, staying at home as much as possible, closure of public buildings and prohibition of social events). For LD1, this was May-June 2020 and for LD2, December 2020–January 2021. For these assessments, participants received an electronic invitation letter and link to an online questionnaire. In total, 44 participants completed all assessments, that is, the imaging assessment at Baseline-17 and three follow-up measurements.

Written informed consent and assent was obtained from participants and parents. The study was approved by the local ethics committee (CMO region Arnhem-Nijmegen, The Netherlands) and the Institutional Review Board of the Faculty of Social Sciences at Radboud University.

### 2.2 Experimental task

During the Go/Nogo Under Threat (GUNT) task, participants had to make fast decisions under threat of shock (Gladwin et al., 2016). They were presented with one of two opponent avatars standing in the center of a parking garage. An armed policeman was featured in the background on the left or right side of the screen (alternating per block). The participant also had a view of his/her own “in task” hands holding a gun. The trial began with one of two randomly presented opponents (the cue). The opponents were visually distinct and signaled the preparation for action. One of the opponents was always associated with electric stimulation (high threat cue) while the other never was; this was counterbalanced across participants. After a varied preparation period (short: 500–1500 ms, middle: 1500–5000 ms, slow: 5000–7000 ms), the opponent could either draw a gun or pull out a mobile phone (stimulus). An armed opponent signaled the participant to shoot, whereas an unarmed opponent (holding a mobile phone), an inhibitory response. Participants were instructed to fire their gun as quickly as possible, before the opponent shot them first (i.e., within the response window) and received visual feedback that they shot the opponent. If participants fired too early (before the gun draw) or shot an opponent holding a phone (i.e., false alarms), they were punished by the policeman standing in the back of the garage (i.e., visual feedback of being shot). If they were too slow, they were shot by the opponent. The visual feedback of being shot was combined with an electric shock only for the highly threatening opponent and not for the low threat opponent. The frequency of aversive stimulations was kept consistent during the task and between participants by titrating the response window to the participant's performance so that they would be shot on approximately 50% of the trails. Electric stimulation was set to an unpleasant, but not painful, level with a standard work-up procedure (Klumpers et al., 2010). The task was run on a PC using Presentation software (http://www.neurobs.com) and was presented on the center of the screen, which participants could view via a mirror above their heads.

The task-procedure consisted of three phases: introduction, training, and measurement. During the introduction phase (4 trials), participants learned which opponent was highly threatening and paired with an electric shock (a shock was always associated with one of the opponents, regardless of shooting performance). In the training phase, participants performed 2 blocks, 90 trials each. The length of the preparation period was distributed such that 80% were short preparation intervals, 10% were middle and 10% slow. The inter-trial interval (ITI) was 400–600 ms. The training phase was included to establish low and high threat stimulus – response associations and differentiate a physiological response between high and low threating trails based on previous studies with this task (Gladwin et al., 2016; Hashemi et al., 2019). The measurement phase consisted of 4 blocks, 30 trials each. To acquire a sufficient number of trials for which the time course of preparation-related freezing responses could be analyzed, 60% of the preparation intervals were slow. Short and middle intervals were also included to ensure that the moment of ‘attack’ was unpredicted. The ITI varied between 50% fast intervals (5000–6000 ms), 30% middle (6000–7000 ms), and 20% slow (7000–8000 ms).

After the task, participants filled in a questionnaire about their subjective responses to the task on a nine-point Likert scale. Using self-assessment manikins, they indicated how they experienced the opponents’ valence (1-pleasant, 9-unpleasant), arousal (1-agitated, 9-calm), and dominance (1-controlled, 9-controlling) (Bradley and Lang, 1994). To assess awareness of experimental contingencies, participants were asked which of the two opponents was associated with a shock and how confident they were about that (1-not sure at all, 9-absolutely sure), how motivated they were to shoot each opponent (1-not motivated, 9-very motivated), and how they experienced the shock at the beginning and end of the task (1-unpleasant, 9-pleasant).

### 2.3 Procedure

The GUNT task was part of a larger assessment carried out within the NLS measurement wave at Baseline-17. At the beginning of the protocol, saliva samples were collected. Next, participants completed an unrelated fMRI task in the scanner (20 min) and an anatomical scan (5 min). A short break (∼15 min) followed during which time the second saliva sample was taken. A finger pulse plethysmography was attached to the left index finger to measure heart rate. Standard Ag/AgCl electrodes were attached to the middle and ring finger to administer shocks via a Maxtens 2000 stimulator. The shocker was individually calibrated (4 min) and participants were familiarized with the setup of the task in the introduction phase (4 min), immediately followed by the training and assessment phases of the task (35 min).

### 2.4 Mental health

The Symptom Check List-90-Revised (SCL-90-R; Arrindell and Ettema, 2003) was used to assess participants’ self-reported mental health on a 5-point scale (1 – Not at all, 5 – A lot). An overall total score was computed for psychological distress, as well as for the anxiety and depression subscales. Missing values on the item level were imputed based on the average of the remaining items of a given scale. Paired sample *t*-tests examining whether mental health problems differed before compared to during the COVID-19 lockdown periods did not show statistically significant differences, similar to previously reported findings in the full NLS sample (for details see van den Berg et al., 2021). The exception to this was psychological distress, which significantly declined during young adulthood in the current subsample, *t*(55) = 2.13, *p* = .038. Of note, the percentage of participants scoring at clinical levels increased by the second lockdown compared to the previous lockdown as well as both baseline assessments.

To predict changes in mental health symptoms from Baseline-17, a difference score was computed between Baseline-20 – Baseline-17 and LD2 – LD1 with positive scores reflecting an increase in symptoms over time. The first two assessments (Baseline-17 and Baseline-20) span a transition period – young adulthood, and as such provide an estimate of base-rate symptom changes during this developmental phase. Increases in symptoms between LD1 and LD2 capture effects of chronic stress related to COVID-19 lockdown, while measurements at LD1 and LD2 capture acute stress responses. A reduction (or lack of increase) in symptoms between LD1 and LD2 captures stressor resilience, as a dynamic process and not merely the lack of symptoms at a given timepoint.

We additionally assessed self-reported burden related to the COVID-19 lockdowns. Participants rated on a 100-point scale (0 - No impact, 100 - A lot of impact), the impact of COVID-19 on their daily lives (i.e., “What is the general impact of the COVID-19 pandemic on your daily life?”). A paired sample *t*-test demonstrated a significant increase in stress burden from the first to second lockdowns, *t*(43) = 2.29 *p* = .027. We therefore included COVID-19 stress burden as a control variable in subsequent analyses predicting mental health. Because COVID-19 stress burden at LD1 was correlated with levels at LD2 (*r* = 0.52, *p* < .001), a sum score was computed. Descriptive information for mental health and stress burden is presented in Table 1.

**Table 1:** Descriptive information for mental health and stress-related symptoms.

	Age (years)	Mean	*SD*	Min	Max	clinical range
*Baseline-17* (*n* = 64/61^)	17.20 (0.17)					
Psychological distress		146.4	47.2	92	288	19.7%
Anxiety		15.3	5.6	10	37	8.2%
Depression		27.2	11.5	16	70	16.4%

*Baseline-20* (*n* = 58)	20.56 (0.13)					
Psychological distress		133.8	37.6	92	319	6.9%
Anxiety		14.2	5.1	10	41	5.2%
Depression		24.9	8.5	16	49	12.1%

*COVID-19 LD1* (*n* = 51)	22.67 (0.20)					
Psychological distress		133.4	33.4	92	225	11.8%
Anxiety		14.0	4.5	10	34	5.9%
Depression		26.1	10.5	16	61	11.8%
COVID-19 stress burden		65.0	22.3	20	100	

*COVID-19 LD2* (*n* = 52)	23.26 (0.21)					
Psychological distress		135.1	37.8	90	253	9.6%
Anxiety		15.0	6.1	10	34	13.5%
Depression		27.4	10.7	16	64	23.1%
COVID-19 stress burden		71.8	17.3	25	100	

Notes: ^64 participants took part in the fMRI assessment at Baseline-17. Questionnaire data is available from 61 participants.

### 2.5 Early-life stress

Early-life events were assessed via parent report at 15 months, 28 months, and 5 years. Parents indicated whether or not their child had experienced a number of life events in the previous 12–24 months that are thought to have an aversive influence on children's development, e.g., illness, divorce, hospitalization of a parent (Gersten et al., 1977). The scale included items from the Life Experiences Survey (Sarason et al., 1978) and the Life Events Scale for Children (Coddington, 1972). This measure was used previously as an index of stressful early-life experiences in this longitudinal study (Niermann et al., 2015; Smeekens et al., 2007; Tyborowska et al., 2018). Consistent with these earlier studies, a total score was calculated indicating the total number of negative life events that took place until age 5.

### 2.6 Pubertal development measures

Following the procedure of Tyborowska et al. (2016), saliva samples for testosterone measures were collected into Salicap (IBL) containers by passive drool in duplicate, 2 hours apart (for details see Supplementary Material S1). Testosterone values between the first and second measurement remained consistent for males, *t*(35) = 1.534, *p* = .134, and females, *t*(27) = 1.408, *p* = .171. Testosterone levels from the first measurement were used for subsequent analyses following (Kaldewaij et al., 2019; Tyborowska et al., 2016; Volman et al., 2011). In addition, pubertal development (secondary sexual characteristics) was assessed with the self-report Pubertal Development Scale (Petersen et al., 1988). For descriptive information see Supplementary Material Table S1.

### 2.7 Heart rate analysis

To assess freezing-related bradycardia in anticipation of high (shock) versus low threat, heart rate data was preprocessed with Matlab2015a and analyzed with SPSS 23. Raw data was down-sampled to 250 Hz and passed through a Butterworth band-pass filter (0.5–10 Hz). An automated peak detection algorithm (developed in-house) was used to assess heart rate. Each trial was then visually inspected and peak detection was manually corrected if required. Trials of at least 5.5 s with correct responses and without electrical stimulation were included in the analysis. Trials with shocks and wrong responses were excluded because they coincided with the switch to action phase. Additionally, the first trial of each block as well as trials with an insufficient signal-to-noise ratio (i.e., where peaks were non-detectable, e.g., due to movement artefacts) were discarded from the analysis. Participants had to have a minimum of 14 remaining trials per condition to be included in the analysis, resulting in datasets of 43 participants for the heart rate analysis. The high exclusion rate (*n* = 21) is likely the result of movement and (scanner) sequence-related artefacts on the finger-clips used to measure HR. Changes in heart rate during the preparation period were calculated in beats-per-minute (BPM) relative to a baseline period of 1 s before event onset.

Because we expected freezing-related bradycardia during the preparation phase, the analysis was time-locked to the cue. Changes in BPM were calculated between 3 and 6 s relative to the baseline period (before cue onset) to exclude orienting effects of freeze (Hagenaars et al., 2014b). Time windows were separated into half-second bins per condition (Graham, 1978) and entered into a repeated measures ANOVA with cue (low vs. high threat) and time (8 time windows centered around the following time points: 3.0 s, 3.5 s, 4.0 s, 4.5 s, 5.0 s, 5.5 s, 6.0 s, 6.5 s post cue) as within-subject factors. Differences in heart rate responses were analyzed in the 3–6.5 s time window base on previously reported dynamics of threat-induced bradycardia and freezing response (Castegnetti et al., 2016; Hagenaars et al., 2014a). Sex was included as a between-subjects factor based on previous reports for this sample (Tyborowska et al., 2016). Planned paired sample *t*-tests were performed to test main effects of threat per time point. The assumption of sphericity was violated for the main effect of time, χ^2^(27) = 360.3, *p* < .001, and the cue × time interaction, χ^2^(27) = 259.2, p < .001), based on Mauchly's test. Therefore, degrees of freedom were corrected using Greenhouse-Geisser estimates (ε = 0.28 for main effect of time and ε = 0.41 for cue × time interaction).

### 2.8 Behavioral analysis

Behavioral data was analyzed with Matlab2015a and SPSS 23. Reaction time (RT) analysis included correct responses during the response window (200–500 ms). Trials in which participants were shot due to the titration were discarded from the analysis. A paired sample *t*-test examined differences in RT between high and low threat trials. A three-way repeated-measures ANOVA with factors cue (high, low), draw (shoot, withhold), and sex (boys, girls) was conducted on accuracy with standardized (per sex) testosterone levels as a covariate. Planned paired-sample *t*-tests compared the effect of high vs. low threat. The α level was set at *p* < .05. To predict stress symptom changes during the COVID-19 pandemic, change scores between LD2-LD1 for anxiety symptoms and COVID-19 stress burden were entered as additional covariates in this ANOVA.

### 2.9 fMRI acquisition

The fMRI data were acquired on a Siemens 3 T TRIO and PRISMA MRI scanners (at same testing site) using a 32-channel coil. Functional scans were acquired using a multiecho echoplanar imaging (EPI) sequence (TRIO TR = 1730 ms; PRISMA TR = 1740 ms; TRIO TE = 11 ms, 24.76 ms; PRISMA TE = 11 ms, 25 ms; flip angle 90°; 37 transversal slices; 3.3 × 3.3 × 3.0 mm voxels; FOV = 212 mm). This type of parallel imaging technique significantly reduces the echo train length, reducing motion artefacts, image distortion, and improving BOLD sensitivity (especially in brain regions typically compromised by a single short TE). This also improves coregistration of functional and anatomical data (Poser et al., 2006). Our data was acquired on two different scanners due to an update of the resonance magnetic system during data collection at the testing site. As a result, 41 participants (out of 64) were scanned on the PRISMA system. T1-weighted images were acquired using an MPRAGE sequence (TR = 2300 ms; TE = 3.03 ms; 192 sagittal slices; 1.0 × 1.0 × 1.0 mm voxels; FOV = 256 mm).

### 2.10 fMRI preprocessing

Functional data were preprocessed and analyzed using the Matlab toolbox SPM12 [Statistical Parametric Mapping (www.fil.ion.ucl.uk/spm)]. The multiecho sequence acquired two echoes per volume at every point in time. The first four volumes of each echo were discarded to control for T1 equilibration effects. Head motion parameters were estimated based on the first echo using a least-squares approach with six rigid-body transformation parameters (translations, rotations) and copied to the second echo. The two echoes were combined into a (echo-time weighted) single volume. Next, the time courses of each voxel were realigned to the middle slice (slice 18) to correct for time differences in acquisition. The T1 anatomical image was coregistered to the mean of the functional images. Using the “Segment” tool (SPM12), the T1 anatomical image was segmented into grey matter, white matter, and CSF. The fMRI time series was normalized and smoothed using a group-specific template based on segmented, grey matter images (T1-weighted). Diffeomorphic anatomical registration through exponentiated lie algebra (DARTEL; Ashburner, 2007) was used for inter-subject registration of the grey matter images. The fMRI time series was then transformed and resampled at an isotropic voxel size of 2 mm, resulting in spatially normalized, Jacobian scaled, and smoothed (8 mm FWHM Gaussian kernel) images.

### 2.11 fMRI analysis

The fMRI time series were analyzed in an event-related design within the general linear model, with the aim to identify brain circuits associated with freezing responses (preparation period) and the switch to action under threat. The first two fast paced practice blocks were excluded from the analysis. Vectors for the low and high threat conditions describing the onset of the cue, shooting, and withhold responses were modeled as stick functions and the preparation period as a boxcar function. Additional regressors separately modeled incorrect responses (shooting too soon, too late or false alarms), shock and button presses. Regressors were convolved with a canonical hemodynamic response function resulting in 8 main task regressors (and 3 additional regressors) in the SPM multiple regression analysis.

Head movement-related effects were modeled using six motion parameters estimated with the spatial realignment procedure. Time courses of signal intensities of white matter, CSF, and the portion of the MR image outside the skull were included as three additional regressors. Inspection of the fMRI signal acquired on the PRISMA reflected sudden slice specific fluctuations in signal intensity too fast to be blood oxygen level dependent (BOLD). We therefore included for all subjects, slice-specific regressors modeling the global signal intensity per slice to ensure that these differences did not bias results. The fMRI time series were high-pass filtered (128 s cutoff), and a first-order autoregressive model was used to account for temporal autocorrelation.

Contrast images for the effects of interest were generated per subject and entered into a group-level random effects analysis using one-sample t-tests. Sex and log-transformed, standardized per sex testosterone levels were included as covariates (Tyborowska et al., 2016). To control for the acquisition of data on different machines, a scanner regressor was included as a covariate of no interest. Following previous analyses (Hashemi et al., 2019), we tested for the effect of high vs. low threat during preparation and switch to action. In addition to whole-brain analyses, we tested for region of interest (ROI) effects within the PAG and amygdala with respect to freezing-related action preparation. During the switch to action, we additionally tested for ROI effects in the PAG and perigenual anterior cingulate cortex (pgACC). The same bilateral PAG mask was used as in Hashemi et al. (2019). The bilateral amygdala mask was taken from the Anatomical Automatic Labeling (AAL) atlas. An anatomical mask of the pgACC was created from the Brainnetome Atlas (Fan et al., 2016; http://atlas.brainnetome.org/bnatlas.html) and was comprised of the parcellation of the subgenual and pregenual region of area 32 (ACC).

To predict mental health outcomes during the COVID-19 pandemic, separate models were run for each mental health symptom category (psychological distress, anxiety, depression) and included two additional regressors with change scores between Baseline-20 - Baseline-17 and LD2-LD1. In a follow-up analysis, separate models for acute anxiety symptom levels at LD1 and LD2 were investigated. For control analyses, the cumulative score of COVID-19 stress burden was added as a regressor to the mental health change models. For acute anxiety symptom models, the COVID-19 stress burden level at the corresponding lockdown was added to the model. In a final step, significant effects were controlled for early-life events.

For all models, whole-brain analyses were based on a cluster-forming threshold of *p* < .001 (uncorrected) and inferences were made at the cluster level (*p*_*FWE*_ < .05). A small volume correction (SVC; *p* < .05) was applied to ROI analyses and inferences were made at the voxel-level (*p*_*FWE*_ < .05). Anatomical inference was drawn by superimposing the SPM images on a standard SPM single-subject T1 template. Brodmann areas (BA) were assigned based on the MRIcron template (http://people.cas.sc.edu/rorden/mricron/index.html).

#### 2.11.1 Functional connectivity analysis

Following the fMRI results, we tested interregional connectivity in the neural circuit involved in preparatory freezing responses. We therefore performed two separate psychophysical interaction (PPI) analyses (Friston et al., 1997) – with the right and left amygdalae as seed regions during high versus low threat preparation intervals. Testosterone levels (log-transformed and standardized per sex) and sex were included as covariates. Voxels included in the seed region were selected for each participant based on a sphere (8 mm radius) around the peak voxel of the group-level activation cluster (left amygdala model MNI coordinates: -22, 0, -12; right amygdala model MNI coordinates: 24, 2, -12). For the PPI, contrasts were generated between the seed region time courses and high versus low threat conditions. These participant-specific contrast images and corresponding regressors for testosterone levels and sex, were entered into a multiple regression analysis. In addition to whole-brain analysis, we tested for ROI effects in the bilateral PAG with a mask taken from Hashemi et al. (2019). A follow-up PPI analysis was conducted on the left amygdala seed during high threat vs. baseline preparation intervals, following the same afore-mentioned procedure.

## 3 Results

### 3.1 fMRI results

#### 3.1.1 Main task effects

**Fig. 2:**
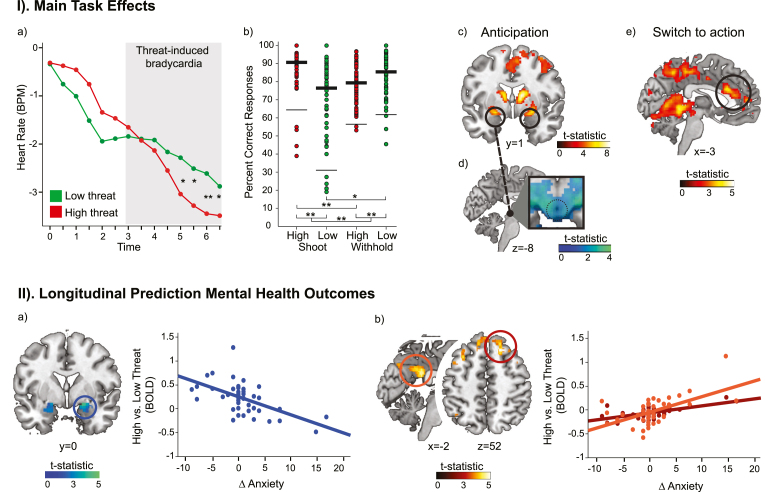
Physiological, behavioral, and neural responses during the GUNT task and longitudinal associations with mental health outcomes during COVID-19. I). Main task effects qualify high versus low threat responses. High threat enhances anticipatory freezing and the tendency to shoot (versus withhold): a) Average heart rate in beats per minute (BPM) during the preparation interval showed increased bradycardia in the high (red) versus the low (green) threat condition. b) During the high threat (vs. low) condition, participants were less accurate in withholding their responses, but also more accurate when shooting was required. Task-related activity during the anticipation phase for high versus low threat c) in the bilateral amygdala and d) negative functional connectivity between the left amygdala and PAG. e) Increased activity in the perigenual anterior cingulate cortex during the switch to shooting responses. Bars show SD = 2; dots represent individual means per participant. Asterisks indicate pairwise significance. Statistical maps thresholded at *p* < .001 uncorrected (c) and *p* < .05 uncorrected (d–e) for display purposes. **p < .001, *p < .01. II). Prediction of COVID-19 mental health outcomes. a) Higher amygdala activity for high (versus low) threat during the anticipation phase was associated with a decrease in anxiety symptoms between the first and second lockdown. b) Higher anticipatory threat-related activity of the posterior cingulate cortex and superior frontal gyrus was related to an increase in anxiety symptoms. Statistical maps thresholded at *p* < .001 uncorrected (b) and *p* < .05 uncorrected (a) for display purposes. (For interpretation of the references to colour in this figure legend, the reader is referred to the Web version of this article.)

First, we examined neural circuits active during the anticipation phase in high versus low threat trials. Significant whole-brain activation was found in subcortical regions associated with threat appraisal and action preparation during the anticipation period, namely the amygdala, insula (local maximum: -20, 2, -10; *p*_*FWE*_ < .001), caudate, thalamus, putamen (local maximum: 12, 0, 12, *p*_*FWE*_ < .001) as well as middle prefrontal cortex regions (Fig. 2-Ic; for all effects see Table 2). The PAG (local maximum: 10, 6, 0; *p*_*FWE*_ < .001) was activated during the anticipation phase, but this was not specific to the high threat condition (Supplementary Material Table S2).

**Table 2:** Significant clusters during the anticipation interval and switch phase.

				MNI coordinates				
Anatomical region	Side	BA	*k*	x	y	z	*p*	*t*
*Anticipation Interval - High vs. Low Threat*								
Caudate/Thalamus/Putamen	R		2671	12	0	12	<.001	7.89
Amygdala/Insula	L	48	627	−20	2	−10	<.001	6.03
Amygdala	R	34		24	2	−12	<.001^a^	5.88
Middle cingulate cortex	L/R	24/32	1266	−2	6	44	<.001	5.90
Middle frontal gyrus	R	6	177	36	0	60	.014	4.57
*Shoot vs. Withhold* × *High vs. Low Threat*								
Cerebellum	R	27	257	2	−44	−4	.002	4.31
*Shoot High vs. Low Threat*								
pgACC	R/L	24		0	26	22	.047^a^	3.93
Cerebellum	L		138	2	−44	−4	.039	4.63
*Shoot vs. Withhold*								
Cerebellum	R		257	2	−44	−4	.002	4.31

Note: BA, Brodmann Area; *k*, number of voxels in a cluster; *p*, FWE-corrected cluster-level value; *t*, t-statistic at the peak voxel; R, right; L, left; pgACC, perigenual anterior cingulate cortex.

^a^SVC *p*_*FWE*_ peak voxel statistic in anatomically defined area.

We also aimed to identify regions involved in the switch from defensive preparation to action under threat. Following the previous study (Hashemi et al., 2019), the pgACC (0, 26, 22; SVC *p*_*FWE*_ = .047) was significantly activated for high vs. low threat shooting responses (Fig. 2-Ie; for all effects see Table 2).

##### 3.1.1.1 Functional connectivity

To assess changes in coupling between the amygdala and the PAG during the preparation interval, we conducted a psychophysiological interaction analysis, with the left and right amygdala (separately) as the seed region and high vs. low threat as a psychological factor. An ROI analysis on the PAG indicated negative connectivity between the left amygdala and the left PAG (MNI coordinates: -4, -26, -8; SVC *p*_*FWE*_ = .047) as a function of high threat (Fig. 2-Id). Post hoc exploration of the nature of this effect by means of an ROI analysis on the PAG (seed left amygdala) for high threat versus baseline as a psychological factor, confirmed that this activation was due to increased negative connectivity during threat (MNI coordinates: -4, -26, -8; SVC *p*_*FWE*_ = .016; MNI coordinates: 6, -26, -10; SVC *p*_*FWE*_ = .044). There was no such effect for the right amygdala seed region. Consistent with the main task analysis, testosterone levels were included as a covariate in the analysis, but did not reveal significant effects between our specified ROIs (amygdala, PAG, pgACC).

#### 3.1.2 Longitudinal prediction of mental health symptoms

Critically, we investigated whether baseline threat-anticipatory task effects, particularly in the amygdala and PAG, could predict COVID-19 related changes in mental health outcomes – with respect to general psychological distress, as well as specifically for anxiety and depression. In post hoc tests, we explored whether task activity predicted acute effects at LD1 and LD2 for anxiety symptoms.

In line with the adaptive role of preparatory threat responses during an active coping paradigm, higher amygdala activity for high (versus low) threat was associated with relative decreases in anxiety symptoms between LD1 and LD2 (Fig. 2-IIa). Less differentiation in amygdala activity to high versus low threat trials, or higher amygdala activity for low threat (versus high threat) was associated with increases in anxiety symptoms between the two lockdown periods. Follow-up analyses showed that this effect was driven by higher amygdala threat-anticipatory activity related to lower levels of anxiety symptoms at LD2, but not LD1.

In contrast to our expectations, PAG activity did not predict symptom changes. However, higher post cingulate cortex (PCC) and superior frontal gyrus (SFG) activity was related to an increase in anxiety symptoms during the lockdowns (Fig. 2-IIb). Higher inferior frontal gyrus (IFG) activity was specifically related to more anxiety symptoms at LD2. With respect to the other symptom measures, decreased mid cingulate activation was associated with psychological distress increases. No significant effects were found for changes in depressive symptoms or any symptom changes between the two baseline measures. All predictive effects were specific for the threat-anticipation phase; the ‘switch to action phase’ did not significantly predict symptom changes. Table 3 contains all significant effects.

**Table 3:** Significant clusters during the anticipation interval related to changes between LD2-LD1 and acute effects at LD1 and LD2.

					MNI coordinates				
	Anatomical region	Side	BA	*k*	x	y	z	*p*	*t*
Δ Psychological Distress	*Increased Activity High vs. Low Threat*								
	–								
	*Decreased Activity High vs. Low Threat*								
	Mid cingulate cortex/WM	R	24	169	18	10	34	.011	4.50
Δ Anxiety	*Increased Activity High vs. Low Threat*								
	Middle occipital gyrus	R	7/19	495	38	−66	34	<.001	5.15
	SFG	R	8	126	18	26	52	.039	5.14
	Middle temporal gyrus	L	21	179	−64	−30	−8	.008	4.76
	Post cingulate cortex	L	26	348	−2	−42	26	<.001	4.70
	Superior fronto-medial gyrus	L	8/9	127	−8	40	50	.038	4.68
	Inferior parietal cortex	L	7	330	−40	−60	56	<.001	4.29
	*Decreased Activity High vs. Low Threat*								
	Amygdala	R	34		20	0	−12	.005^a^	4.51
LD2 - Anxiety	*Increased Activity High vs. Low Threat*								
	IFG/WM	R	48/45	127	28	32	8	.039	5.42
	*Decreased Activity High vs. Low Threat*								
	Thalamus/amygdala	R	34	295	8	−6	−2	<.001	5.87
	Rolandic operculum/supramarginal gyrus	L	48	323	−44	−16	20	<.001	5.56

Note: BA, Brodmann Area; *k*, number of voxels in a cluster; *p*, FWE-corrected cluster-level value; *t*, t-statistic at the peak voxel; R, right; L, left; WM, white matter; SFG, superior frontal gyrus; IFG, inferior frontal gyrus.

^a^SVC *p*_*F*__*WE*_ peak voxel statistic in anatomically defined area.

Effects of interest remained significant when controlling for COVID-19 stress burden (Supplementary Material Tables S3 and S4). In a final step, amygdala, IFG, SFG, and PCC effects were extracted and controlled for prospectively assessed early-life stress. All associations remained significant (all *p*'s ≤ .005, *r* > 0.4).

### 3.2 Behavioral and psychophysiological manipulation checks

#### 3.2.1 Main task behavioral analysis

Based on previous work in adults using the same task (Hashemi et al., 2019), we expected that the threat of shock would increase the speed and tendency to shoot. A significant cue × draw interaction for accuracy, *F*(1, 61) = 50.61, *p* < .001, confirmed this for the tendency to shoot (Fig. 2-Ib). If participants were required to withhold their responses, they were less accurate on high versus low threat trials, *t*(63) = −4.005, *p* < .001. However, if required to shoot, they made less mistakes on high threat trials, *t*(63) = 5.802, *p* < .001. Within the high threat condition, participants were also better at shooting than withholding, *t*(63) = 4.938, *p* < .001, whereas the opposite pattern was true for low threat trials, *t*(63) = −3.101, *p* = .003. The accuracy effect did not interact with testosterone levels, *F*(1, 61) = 0.31, *p* = .861, or sex, *F*(1, 61) = 0.383, *p* = .539. There was also a main effect of cue, *F*(1, 61) = 6.244, *p* = .015, indicating overall higher accuracy on high threat trials. There was no significant difference in RT for high and low threat trials, *t*(63) = −1.326, *p* = .19.

##### 3.2.1.1 Longitudinal prediction of mental health symptoms

Task accuracy effects (cue × draw interaction) were not significantly associated with changes in anxiety symptoms, *F*(1, 40) = 0.626, *p* = .434. The main effect of cue, *F*(1, 40) = 5.9, *p* = .02, and the cue × draw interaction, *F*(1, 40) = 33.17, *p* < .001, remained significant in this model.

#### 3.2.2 Heart rate response

A significant main effect of time, *F*(1.95, 79.97) = 18.767, *p* < .001, indicated a decrease in heart rate responses during the preparation phase. A significant cue × time interaction, *F*(2.85, 116.89) = 5.011, *p* = .003, showed that this decrease was more pronounced in the high threat condition (Fig. 2-Ia). This was confirmed by post hoc *t*-tests at time points 5.0 s, *t*(42) = 2.38, *p* = .022, 5.5 s, *t*(42) = 2.67, *p* = .011, 6.0 s, *t*(42) = 3.27, *p* = .002, and 6.5 s, *t*(42) = 2.36, *p* = .023, post cue. These findings replicate previous findings in humans showing that anticipation of threat is related to robust fear bradycardia (Castegnetti et al., 2016; Hashemi et al., 2019; Klaassen et al., 2021).

## 4 Discussion

The goal of the study was two-fold. First, we aimed to isolate neural defensive circuits and physiological responses related to freezing and the subsequent switch to action in a late adolescent sample. Second, we aimed to evaluate whether these acute threat-related neural defensive responses provide resilience and vulnerability markers in the development of chronic stress-related symptoms. The outbreak of the COVID-19 pandemic, with ensuing lockdown periods, presented the opportunity to investigate the predictive value of these neural markers to a stressful event affecting the entire population. Assessing these factors in an existing longitudinal sample offers the rare opportunity to control for prospectively quantified early-life stressors.

In line with previous work in adults with the GUNT task, during the anticipation phase, we observed increased bradycardia under threat of shock, indicative of a freezing response. This response was accompanied by increased activity in threat evaluation and action preparation regions, including the thalamus, caudate, insula, amygdala (Hashemi et al., 2019). During the subsequent transition to fight, the pgACC was activated. Critically, amygdala and prefrontal activity during the anticipation phase was predictive of changes in anxiety symptoms between the COVID-19 lockdowns. Specifically, higher amygdala, but lower PCC and SFG activity during high (versus low) threat trials was associated with a decrease in anxiety levels. Interestingly, and in line with Weissman et al (2021), higher amygdala responses to low threat versus high threat was related to an *increase* in anxiety symptoms, and *less resilience* to COVID-19 lockdown-related stress. These effects occurred over and above any symptom changes occurring between age 17 and age 20, experienced COVID-19 stress burden, and early-life stress (0–5 years). Overall, the findings of this prospective longitudinal study support the notion that neural markers of threat reactivity, assessed in the context of action-preparatory threat-coping, may signal resilience against the development of psychopathology in stressful contexts.

### 4.1 Neural correlates of anticipatory threat and switch to action

Many of the neural, behavioral, and physiological findings of the present study are in line with those reported in adults on the GUNT task (Gladwin et al., 2016; Hashemi et al., 2019; Klaassen et al., 2021). Similar to adults, we found increased bradycardia in the high threat condition and more shooting reactions during the switch to action, even when shooting had to be withheld, indicative of a ‘trigger-prone’ reaction profile. Activation patterns in the thalamus, striatum, and cingulate regions during the preparation interval replicated previous fMRI work using this task (Hashemi et al., 2019). Our results extend previous studies by showing that also during late adolescence, anticipatory activity in the amgydala-PAG circuit facilitates action preparation and highlight the role of the pgACC in switching to action. This also supports the notion that the PAG-amygdala-mPFC circuit plays a critical role in facilitating the neural switch from passive to active defense reactions (Gozzi et al., 2010; Tovote et al., 2015).

Although the psychophysiological and behavioral effects found in adolescents are similar to those in adults, in adolescence we found specific threat-related amygdala activity, but lack of PAG activation, during the anticipation phase. This was concurrent with negative amygdala-PAG connectivity on high (versus low) threat conditions - while adults exhibited positive connectivity between these areas irrespective of threat (Hashemi et al., 2019). In light of the increased threat-induced amygdala activity, we speculate that the negative pattern of amygdala-PAG connectivity found here may signal the amygdala's inhibition of the dlPAG during freeze (Keay and Bandler, 2001; Tovote et al., 2016; Walker and Carrive, 2003).

The lack of threat-specific PAG activation during action preparation as well as absence of PAG and amygdala activity during the switch to action does fit with studies in mice that show a non-linear pattern in amygdala-dependent fear responses - with blunted responses appearing in adolescent mice (Pattwell et al., 2013). The central nucleus of the amygdala, together with the mPFC, are responsible for shifting responses to a threatening stimulus from freeze to action (Gozzi et al., 2010; Moscarello and LeDoux, 2013). In adolescent mice, diminished contextual fear expression, i.e., less freeze reactions, has been related to lower threat-related output of the central nucleus as a result of immature mPFC – limbic circuitry (Pattwell et al., 2011; Pattwell et al., 2013). These findings are also in line with reported increases in mPFC innervation of the amygdala with age and increased pruning of mPFC - amygdala inputs during late adolescence (Zimmermann et al., 2019).

### 4.2 Neural predictors of mental health

Heightened action-preparatory amygdala activity during high (versus low) threat was associated with resilience (i.e., less increase in anxiety symptoms between the first and second lockdown periods). This finding contrasts with previous work reporting heightened amygdala activity, particularly in response to threat, as a neural risk marker for psychopathology (Fareri and Tottenham, 2016; McLaughlin et al., 2014; Shackman et al., 2017). The results of the current study are in line, however, with the adaptive role of threat anticipatory activity (Funkhouser et al., 2022; Roelofs and Dayan, 2022), with particularly blunted freezing responses linked to vulnerability for internalizing symptom development across adolescence and young adulthood (Held et al., 2022; Niermann et al., 2018). In fact, a recent study in early and middle adolescents showed that lower amygdala responses to angry faces – and consequently higher activity to neutral faces - was related to more internalizing symptoms during the COVID-19 pandemic (Weissman et al., 2021). The authors suggest that higher amygdala reactivity to neutral compared to threatening cues may reflect uncertainty in the interpretation of ambiguous neutral faces, and hence signal a vulnerability for psychopathology under chronic and uncertain stressful conditions. Similarly in the current study, the somewhat atypical pattern of lower amygdala activation for *high threat* trials – or higher amygdala reactivity to *low threat* trials - was related to increases in anxiety symptoms across the COVID-19 pandemic. In other words, less typical adult-like differentiation in amygdala activity to high compared to low threat trials may likewise reflect a greater risk factor for symptom development.

The amygdala, as part of the neural circuit responsible for defensive behavior, should be activated in response to threat, particularly when there is the possibility to actively cope with a stressor (Gu et al., 2020; Tovote et al., 2015). Therefore, a balanced amount of reactivity is an adaptive and healthy response to a threat or stressor (Funkhouser et al., 2022). In the same vein, our results suggest that a ‘normative’ engagement of the amygdala during high (versus low) threat may be associated with relatively stronger resilience to the development of stress-related symptomology. Likewise, it can be argued that an under-reactive amygdala to threat (or an over-reactive one to low-threatening stimuli), is the consequence of an inadequately engaged defensive circuit, and as such maladaptive for later periods of heightened stress.

Amygdala hypoactivation during a threatening context specifically predicted acute anxiety levels at the second lockdown. LD2 likely reflects either a second, even higher, spike of symptoms, or a build-up of anxiety levels across the pandemic. Amygdala effects associated with both an increase in symptoms as well as acute levels at LD2 are consistent with cumulative risk models, where the number of stressors or adverse events has an additive impact on mental health (Evans et al., 2013; McLaughlin et al., 2022).

These effects occurred above and beyond known developmental changes occurring between adolescence and young adulthood, reported impact of COVID-19, as well as early-life history (Smith and Pollak, 2021; Zahniser and Conley, 2018). Importantly, amygdala activity was also not related to any changes in affective symptoms (anxiety, general psychological distress, depression) during the period preceding the lockdowns. This supports the notion that threat-induced amygdala reactivity may not be a direct correlate of internalizing symptoms, but rather a more complex marker of underlying resilience or vulnerability during periods of heightened stress (Weissman et al., 2021).

Exploratory analyses showed that higher threat-related PCC and SFG activity was associated with an increase in anxiety symptoms between LD1 and LD2, while IFG activity was related to acute symptom levels at LD2. This is in line with studies in anxious individuals showing hyperactivation in medial prefrontal cortices (Chavanne and Robinson, 2021), particularly during anticipation of uncertain threat (Geng et al., 2018). Increased threat-induced PCC activation, alongside lateral prefrontal and parietal activity, has also been linked to enhanced information uptake during Go/NoGo paradigms (Gorka et al., 2022; Torrisi et al., 2016). Speculatively, overtly engaged sensory processing could signal a vulnerability during periods of chronic stress.

Prefrontal and amygdalar regions were not related to psychological distress nor depressive symptoms during the COVID-19 lockdowns. The PAG was not associated with any symptom changes or acute levels at LD1 or LD2. We also found no associations during the switch to action phase with later symptoms. Our findings therefore point to the predictive value and potentially adaptive role of threat-related activity, specifically during an anticipation period of action preparation for resilience and vulnerability markers.

### 4.3 Interpretational issues

This study had several strengths including extensive and multimodal data (self- and parent-report, physiology, neuroimaging) sampled at four time points – two baseline measurements and two COVID-19 time points. In addition, this study had the possibility to control for pre-COVID stress (even early-life stress until age 5) and symptom levels in a prospective longitudinal fashion. However, some limitations should be considered when interpreting the findings. First, we investigated automatic defense reactions in adolescents using a task previously validated in adults. However, in order to gain insight into developmental effects in mPFC, amygdala, and PAG task activation, future studies should measure within-person changes at multiple time points across adolescence. For example, a second GUNT task at a later age would allow us to trace changes in these responses. In addition, while a narrow age range helps to characterize effects for a specific developmental window, generalizing the findings of this study to other developmental periods (e.g., early or middle adolescence) should be done with caution. Second, the reliability of the predictive inferences may be limited by the moderate sample size of this study. However, that limitation should be weighed against the precision and specificity afforded by the accurate characterization of the developmental profile of each participant, covering 17–24 years of age, and including early-life history from the first 5 years of life. Third, while mental health symptom assessments were taken at multiple timepoints, neuroimaging task data was collected once in late adolescence. Hence, it was not possible to examine associations of mental symptoms with concurrent neural states in young adulthood. As such, we are not able to assess the stability of the threat-related activations over time and whether adolescent brain states predicted anxiety symptoms above and beyond concurrent neural reactivity. Fourth, we used a global index of Covid-19 burden to account for a wide scope of daily life impact caused by the lockdowns. It is possible, however, that specific individual factors (e.g., social support, health, living situation) may have impacted symptom levels on top of the reported Covid-19 burden. Lastly, future studies investigating resilience factors should also consider positive aspects of mental health, and not only reduction of symptoms. Despite these limitations, the current findings contribute to formulating a multidimensional approach to risk and resilience factors for mental health not only during the pandemic, but also in high-stress contexts more broadly.

### 4.4 Conclusions

This prospective longitudinal investigation in adolescents indicated that acute threat-induced amygdala responding during action preparation predicted *resilience* to subsequent stress, whereas heightened amygdala responses to low threat signals predicted *vulnerability* for the development of anxiety symptoms. Our findings indicate that acute threat-evoked neural defense responses, particularly when differentiating responses to threat and safety signals, may provide longitudinal resilience markers against future stressful events.

## Funding

This work was supported by 10.13039/501100000781European Research Council starting and consolidator grants (ERC_StG_313749 and ERC_CoG_772337, awarded to KR) and European Union funded FP7-HEALTH-2013-INNOVATION (602805-2) and Horizon-2020 Research and Innovation (777084) grants. Additionally, the 10.13039/501100003246Netherlands Organisation for Scientific Research supported IT (Vici grant 453-08-002) and HCMN (Research Talent Grant 406-13-022).

## Data and materials availability

Data is available upon request via the Donders Repository (https://data.donders.ru.nl/). Data can be provided by the Donders Institute for Brain, Cognition, and Behaviour pending scientific review and a completed data transfer agreement. Requests for the data should be submitted to AT or KR.

## Author contributions

AT – conceptualization, data curation, formal analysis, investigation, methodology, writing – original draft, writing – review and editing. YvdB – conceptualization, data curation, investigation, writing – review and editing. MH – methodology, writing – review and editing. HN – investigation, funding acquisition, writing – review and editing. AC – conceptualization, funding acquisition, writing – review and editing. IT – conceptualization, methodology, supervision, writing – review and editing. KR – conceptualization, funding acquisition, methodology, supervision, writing – review and editing.

## Declaration of competing interest

The authors declare that they have no known competing financial interests or personal relationships that could have appeared to influence the work reported in this paper.
