# Supplementary material for: Neural patterns of threat response in adolescents predict vulnerability for and resilience against internalizing symptoms during COVID-19 waves
Source: Neuroimage Rep. 2023 May 29;3(3):100177. doi: 10.1016/j.ynirp.2023.100177 (PMC12172782; doi:10.1016/j.ynirp.2023.100177)
Supplement: Multimedia component 1 [file mmc1.docx]

**Supplementary Material**

S1. Testosterone sampling

Saliva samples for testosterone measures were collected into Salicap (IBL) containers by passive drool of ~2ml and stored at ~24°C. Testosterone concentration was measured using a competitive chemiluminescence immunoassay (CLIA) with a sensitivity of 0.0025 ng/mL (IBL), whose intra-assay and inter-assay coefficients are between 10% and 12%. Participants were instructed to refrain from heavy physical exercise and consuming food, cigarettes, and drinks (except water) at least ~1 h before the experiment. Girls were tested outside the menstruation phase of their cycle. Since during adolescence testosterone levels may undergo variations particularly during the night (boys) and early morning (Albertsson-Wikland et al., 1997; Ankarberg & Norjavaara, 1999), it was always sampled after 10:00 A.M and in duplicate, 2 hours apart. The choice of testosterone as a physiological marker of pubertal development in both females and males is supported by previous work (Huang et al., 2012; Shirtcliff et al., 2009; Tyborowska et al., 2016). Because testosterone can act on receptors through its conversion to other steroid hormones, it can have similar effects in females and males (Burger, 2002).

S2. Excessive movement criteria (fMRI)

Participants who moved more than 3mm between scans (n=4) in any X, Y or Z direction were visually inspected. For n=3 participants, translation in Z was =< 4mm and in the X and Y directions < 3mm. The increased movement occurred at the end of the task before the scanner was turned off (n=1) or between blocks (n=2). One participant was excluded due to overall excessive movement.

Table S1. Demographic information: testosterone levels, PDS, and age per sex

|  | Testosterone t1 (pg/ml) | Testosterone t2 (pg/ml) | PDS score | Age (years) |
| --- | --- | --- | --- | --- |
| Males | 123 (63) | 135 (75) | 3.4 (0.5) | 17.21 (0.17) |
| Females | 29 (17) | 26 (15) | 3.5 (0.4) | 17.11 (0.15) |

Values represent the mean (SD). Testosterone t1 and t2 represent the first and second salivary measurement (respectively). Sex refers to sex assigned at birth.

PDS, Pubertal Development Scale.

Table S2. Anticipation phase (high and low threat)

|  |  |  |  | MNI coordinates | | |  |  |
| --- | --- | --- | --- | --- | --- | --- | --- | --- |
| Anatomical region | Side | BA | *k* | x | y | z | *p* | *t* |
| Thalamus, Caudate, Insula, Amygdala, PAG | R/L | 25 | 412680 | 10 | 6 | 0 | <.001 | 16.26 |
| Precentral gyrus | L | 6 | 589 | -38 | -6 | 48 | <.001 | 8.28 |
| Middle / Superior frontal gyrus | R | 10/46 | 1537 | 34 | 46 | 20 | <.001 | 8.13 |
| Precuneus | R/L | 7 | 228 | -8 | -68 | 38 | <.009 | 6.38 |
| Superior parietal lobule | L | 7/40 | 460 | -30 | -50 | 52 | <.001 | 5.98 |
| Inferior parietal lobule | R | 40 | 152 | 30 | -48 | 48 | .05 | 5.66 |
| *Girls>Boys* | | | | | | | | |
| Amygdala | L | 34/48 | 205 | -22 | 4 | -12 | .015 | 4.27 |
| *Negative testosterone modulation* | | | | | | | | |
| Olfactory cortex | L | 25 | 352 | -6 | 20 | -14 | .001 | 5.89 |
| Superior frontal gyrus | L | 9/32 | 157 | -12 | 50 | 32 | .044 | 4.84 |

Note: BA, Brodmann Area; *k*, number of voxels in a cluster; *p*, FWE-corrected cluster-level value; *t*, t-statistic at the peak voxel; R, right; L, left.

Table S3. Significant clusters during the anticipation phase related to changes between LD2-LD1 and acute effects at LD1 and LD2 controlling for Covid-19 stress burden

|  |  |  |  |  | MNI coordinates | | |  |  |
| --- | --- | --- | --- | --- | --- | --- | --- | --- | --- |
|  | Anatomical region | Side | BA | *k* | x | y | z | *p* | *t* |
| Δ Psychological Distress | *Increased activity* |  |  |  |  |  |  |  |  |
|  | - |  |  |  |  |  |  |  |  |
|  | *Decrease activity* |  |  |  |  |  |  |  |  |
|  | Mid cingulate cortex / WM | R | 24 | 169 | 18 | 10 | 34 | .03 | 4.61 |
| Δ Anxiety | *Increased activity* |  |  |  |  |  |  |  |  |
|  | SFG | R | 8 | 140 | 18 | 26 | 52 | .025 | 5.15 |
|  | Middle occipital gyrus | R | 7/19 | 465 | 38 | -66 | 34 | <.001 | 4.95 |
|  | Inferior temporal gyrus/ Middle temporal gyrus | L | 20/21 | 150 | -50 | -38 | -16 | .018 | 4.79 |
|  | Post cingulate cortex | L/R | 26 | 281 | 0 | -42 | 30 | .001 | 4.66 |
|  | Superior fronto-medial gyrus | L | 8/9 | 160 | -8 | 38 | 50 | .013 | 4.50 |
|  | Frontal pole | R | 10 | 330 | 40 | 60 | 0 | .041 | 4.48 |
|  | *Decreased activity* |  |  |  |  |  |  |  |  |
|  | Rolandic operculum / postcentral gyrus | L | 48 | 691 | -52 | 4 | 6 | <.001 | 5.23 |
|  | Amygdala | R | 34 |  | 20 | 0 | -12 | .003^a^ | 4.66 |
| LD2 - Anxiety | *Increased activity* |  |  |  |  |  |  |  |  |
|  | IFG /WM | R | 48/45 | 118 | 28 | 32 | 8 | .052^^^ | 5.26 |
|  | *Decreased activity* |  |  |  |  |  |  |  |  |
|  | Thalamus/ amygdala | R | 34 | 275 | 8 | -6 | -2 | .001 | 5.72 |
|  | Rolandic operculum / supramarginal gyrus | L | 48 | 305 | -44 | -16 | 20 | <.001 | 5.67 |
| Note: BA, Brodmann Area; *k*, number of voxels in a cluster; *p*, FWE-corrected cluster-level value; *t*, t-statistic at the peak voxel; R, right; L, left; WM, white matter; SFG, superior frontal gyrus.  ^a^SVC *p_FWE_* peak voxel statistic in anatomically defined area  ^^^subthreshold effect | | | | | | | | | |

Table S4. Significant clusters during the anticipation phase related to cumulative Covid-19 stress burden during LD1 and LD2 from Anxiety symptom change model

|  |  | |  |  | MNI coordinates | | |  |  |
| --- | --- | --- | --- | --- | --- | --- | --- | --- | --- |
| Anatomical region | | Side | BA | *k* | x | y | z | *p* | *t* |
| *Increased activity* | | | | | | | | | |
| Amygdala / Hippocampus / PHG | | L | 34 | 153 | -30 | -2 | -24 | .016 | 4.65 |
| *Decrease activity* | |  |  |  |  |  |  |  |  |
| Lingual gyrus ^i^ | | R | 17 | 176 | 2 | -88 | -12 | .008 | 5.77 |
| Note: BA, Brodmann Area; *k*, number of voxels in a cluster; *p*, FWE-corrected cluster-level value; *t*, t-statistic at the peak voxel; R, right; L, left; PHG, parahippocampal gyrus.  ^i^ same cluster and peak voxel significant in psychological distress change model | | | | | | | | | |

**References**

Albertsson-Wikland, K., Rosberg, S., Lannering, B., Dunkel, L., Selstam, G., & Norjavaara, E. (1997). Twenty-four-hour profiles of luteinizing hormone, follicle-stimulating hormone, testosterone, and estradiol levels: A semilongitudinal study throughout puberty in healthy boys. *Journal of Clinical Endocrinology and Metabolism*, *82*(2), 541–549. https://doi.org/10.1210/jc.82.2.541

Ankarberg, C., & Norjavaara, E. (1999). Diurnal rhythm of testosterone secretion before and throughout puberty in healthy girls: Correlation with 17-beta estradiol and dehydroepiandrosterone sulfate. *Journal of Clinical Endocrinology and Metabolism*, *84*(3), 975–984. https://doi.org/10.1210/jcem.84.3.5524

Burger, H. G. (2002). Androgen production in women. *Fertility and Sterility*, *77*(4), S3–S5. https://doi.org/10.1016/S0015-0282(02)02985-0

Huang, B., Hillman, J., Biro, F. M., Ding, L., Dorn, L. D., & Susman, E. J. (2012). Correspondence between gonadal steroid hormone concentrations and secondary sexual characteristics assessed by clinicians, adolescents, and parents. *Journal of Research on Adolescence*, *22*(2), 381–391. https://doi.org/10.1111/j.1532-7795.2011.00773.x

Shirtcliff, E. A., Dahl, R. E., & Pollak, S. D. (2009). Pubertal development: Correspondence between hormonal and physical development. *Child Development*, *80*(2), 327–337. https://doi.org/10.1111/j.1467-8624.2009.01263.x

Tyborowska, A., Volman, I., Smeekens, S., Toni, I., & Roelofs, K. (2016). Testosterone during puberty shifts emotional control from pulvinar to anterior prefrontal cortex. *The Journal of Neuroscience*, *36*(23), 6156–6164. https://doi.org/10.1523/JNEUROSCI.3874-15.2016
